# Accuracy of *p53* Codon 72 Polymorphism Status Determined by Multiple Laboratory Methods: A Latent Class Model Analysis

**DOI:** 10.1371/journal.pone.0056430

**Published:** 2013-02-18

**Authors:** Stephen D. Walter, Corinne A. Riddell, Tatiana Rabachini, Luisa L. Villa, Eduardo L. Franco

**Affiliations:** 1 Department of Clinical Epidemiology and Biostatistics, McMaster University, Hamilton, Ontario, Canada; 2 Department of Epidemiology, Biostatistics, and Occupational Health, McGill University, Montreal, Quebec, Canada; 3 Ludwig Institute for Cancer Research, Hospital Alemão Oswaldo Cruz,São Paulo, São Paulo, Brazil; 4 Department of Oncology, McGill University, Montreal, Quebec, Canada; Albert Einstein College of Medicine, United States of America

## Abstract

**Introduction:**

Studies on the association of a polymorphism in codon 72 of the *p53* tumour suppressor gene (rs1042522) with cervical neoplasia have inconsistent results. While several methods for genotyping *p53* exist, they vary in accuracy and are often discrepant.

**Methods:**

We used latent class models (LCM) to examine the accuracy of six methods for *p53* determination, all conducted by the same laboratory. We also examined the association of *p53* with cytological cervical abnormalities, recognising potential test inaccuracy.

**Results:**

Pairwise disagreement between laboratory methods occurred approximately 10% of the time. Given the estimated true p53 status of each woman, we found that each laboratory method is most likely to classify a woman to her correct status. *Arg/Arg* women had the highest risk of squamous intraepithelial lesions (SIL). Test accuracy was independent of cytology. There was no strong evidence for correlations of test errors.

**Discussion:**

Empirical analyses ignore possible laboratory errors, and so are inherently biased, but test accuracy estimated by the LCM approach is unbiased when model assumptions are met. LCM analysis avoids ambiguities arising from empirical test discrepancies, obviating the need to regard any of the methods as a “gold” standard measurement. The methods we presented here to analyse the *p53* data can be applied in many other situations where multiple tests exist, but where none of them is a gold standard.

## Introduction

A polymorphism (rs1042522) in codon 72 of the *p53* tumour suppressor gene (Arg72Pro) has been extensively studied as a risk factor for cervical cancer and its precancerous lesions, but the results have been largely inconsistent [1], [2]. Although a variety of laboratory methods for establishing *p53* status exist, they vary with respect to their reliability, in terms of inherent accuracy and under different assay and specimen conditions. Using more than one method can lead to discrepancies in the estimate of a subject’s *p53* codon 72 genotype, which leads to irresolvable ambiguities about how one should best estimate the association between this genetic marker and cervical neoplasia. A recent editorial [3] has suggested that problems of this type are widespread in genetic testing. In the face of these ambiguities, it is not clear which method (if any) might be preferred to assess this association, or which might provide the best estimate of the disease odds ratio (OR). Furthermore, because empirical estimates of the association ignore the possibility of test errors, they are inevitably subject to bias. By using a latent class model (LCM) that incorporates information from multiple imperfect tests, we can evaluate the accuracy of alternative test methods, and improve the assessment of the association of the genetic marker with disease. In this paper, we develop a LCM approach to this type of data, and illustrate the results from a study of the relation between *p53* polymorphism and disease.

We previously investigated the variation between laboratories in determining *p53* genotype from split samples originating from the same sample of women enrolled in a case-control study [4]. In that work, three international laboratories used the same, standardised laboratory procedure to ascertain the genotype. Discrepancies between laboratory results for the same woman were not infrequent, leading to uncertainty about how to best represent the association of *p53* with cervical cancer. Our analytical approach was to use a LCM [5]–[11], which avoids the need to regard any of the laboratories as a gold standard or in any way superior to the others, but instead takes the inaccuracies of each laboratory into account. The sensitivity and specificity of each laboratory’s data were estimated by the LCM, and the model also adjusted the disease OR for the laboratory measurement errors.

LCMs have been widely used in many areas of medical research for situations in which a gold standard diagnosis of disease cannot be achieved, or where there are problems involved in measuring risk factors. The statistical estimation of the LCM model parameters is based on maximum likelihood, which implies some optimality in the results. Specifically, under a correctly specified model, maximum likelihood estimates are asymptotically unbiased and have maximum precision. The LCM also avoids the ambiguities caused by discrepancies in results that occur when multiple tests are used.

In this paper, we describe several LCMs used to examine the discrepancies between six competing laboratory methods for *p53* determination, all conducted blindly by the same laboratory. The model strategy provides estimates of accuracy for each alternative test method, even though all of them are potentially subject to error. The strength of the method is that, in doing so, we are not required to incorrectly assume that any of the methods represents a preferred or ‘gold’ standard measurement.

As a secondary objective we examine the association of *p53* with cervical abnormalities found on cytology, again taking inaccuracy of the data into account. Finally, we compare the results yielded by the latent class models with the empirical associations. The latter ignore the possibility of laboratory errors; again, this is obviously problematic, because the existence of discrepancies in test results for the same woman indicates that errors do in fact occur, and which therefore bias the empirical OR estimates.

The methods we propose are generalizable to other situations where multiple, imperfect tests exist and when it is unreasonable to assume a gold standard measure.

## Materials and Methods

### Ethics Statement

We used a subset of data from the Ludwig-McGill cohort study of human papillomavirus (HPV) infection and cervical intraepithelial neoplasia [12], [13]. Subjects entered the study only after giving signed informed consent. All of the study procedures and the informed consent were approved by the institutional review boards and ethical committees of the participating institutions: McGill University, Montreal, Quebec, Canada; the University of Toronto, Toronto, Ontario, Canada; and the Ludwig Institute for Cancer Research and the MEVNC clinic, both in São Paulo, Brazil.

### Epidemiological Study

The original study cohort consists of 2528 women aged 18–60 years (median = 33 years) from a maternal and child health program for low income families in Sao Paulo, Brazil. The racial composition of the study was comparable to that in the source population. Most women were of white (European), black, and mixed (*mulata)* ancestry, with less than 2% being of other ancestries. Cervical and blood samples were obtained, together with questionnaires, at the initial enrolment into the study, at follow-up visits every four months during the first year, and then twice yearly thereafter for at least eight years (with questionnaires at annual follow-up returns). This sampling was done on an unselected basis from the population of screened women, and hence is free from genetic selection bias. At the time the specimens were tested for this report there had been 24,545 visits from 2462 women, with a mean of ten visits per subject and 149,184 women months of follow-up (mean = 61 months, median = 76 months). We limited the dataset to women who had at least four out of six possible visits in the first two years of follow-up. More information on the study design and methods for the original study can be found elsewhere [12].

The outcome of interest in the Ludwig McGill study was two-year cumulative risk of squamous intraepithelial lesions (SIL).We chose to exclude women whose worst cytology outcomes were Atypical Squamous Cells of Undetermined Significance (ASCUS) or Atypical Glandular Cells of Undetermined Significance (AGUS), because their results lie somewhere between the case and non-case outcomes used in our analysis. We felt that there would not be a benefit to including ASCUS and AGUS because these are ambiguous cases in which cytology was equivocal. We believe that the best comparison to examine effects of the polymorphism is by contrasting the non-equivocal categories of lesion outcome. The polymorphism in question is not a marker of disease but potentially of susceptibility to HPV and in consequence to disease.

### p53 Codon 72 Genotyping

We assayed DNA samples from 963 women, comprising those with long term follow-up and complete cytological and HPV DNA testing results at the end of 2002, after the exclusions described above. Details of the genotyping methods are described elsewhere [14]. In brief, we amplified a 279 base-pair fragment of exon 4 of *p53* by a polymerase chain reaction protocol (PCR). *P53* codon 72 genotyping of the amplified products was done in all samples by four methods: (i) denaturing high-performance liquid chromatography (DHPLC), (ii) dot blot hybridization (DBH) with sequence-specific oligonucleotide probes for *p53*Arg and *p53Pro*, (iii) restriction fragment length polymorphism (RFLP) analysis after cleavage of the PCR products with the BsaJI endonuclease (RFLP-1) and (iv) with the BstUI endonuclease (RFLP-2). Two additional genotyping assays were also conducted in a subset of 144 women for whom sufficient DNA was available for extended testing. These include direct sequencing (DS) of the exon 4 amplified product and allele-specific PCR (AS-PCR).

Our analysis focused on the first four laboratory methods (DHPLC, DBH, RFLP-1, and RFLP-2). However, other analyses were carried out incorporating the additional data for the subset of women in which the DS and AS-PCR methods were also performed.

No research has been done on the determinants of error for these methods, however a separate paper by our laboratory colleagues describes the relative value of these methods from an assay performance perspective [14]. They each represent a balance between simplicity of the assay versus biological accuracy (i.e., sensitivity and specificity for the nucleotide sequences that identify the genotypes). Other than the usual work done to establish each technique in the laboratory to make sure that they work suitably based on performance standards and appropriate controls, there has been no systematic work on the repeatability of the assays in our lab or, to our knowledge, in other labs. That being said, these techniques represent legitimate alternative ways of analyzing *p53* codon 72 polymorphism (rs1042522).

### Statistical Analysis

We first examined the pattern of disagreements between the results of the alternative test methods. Crude and chance-corrected levels of agreement were calculated using weighted kappa statistics using Cicchetti and Allison weights [15], [16]. In our primary analyses, we considered the genotype results as a variable with 3 levels (*Arg/Arg*, *Arg/Pro*, and *Pro/Pro*). The *Arg/Arg* genotype has been associated with increased risk of cervical neoplasia in many previous studies [1], [2].

In our latent class models, a woman’s true *p53* status is the latent (unobserved) variable, and the probability of being classified into the three possible levels of the *p53* status by each method is estimated for each woman, conditional on her true status. These probabilities are then combined over the various possible levels of the actual status, according to the model specifications; this combination forms the contribution to the overall likelihood of the data from a particular woman. For instance, if the tests are assumed to be independent, the likelihood contribution is made up of a set of products of probabilities of the individual test results, with each set corresponding to the actual *p53* status of a given woman; the sets are summed across levels of actual *p53* status, using weights that correspond to the prevalences of the various *p53* genotypes.

Finally, following standard maximum likelihood methods, the logarithms of the likelihood contributions are summed over study participants, and this sum is maximised numerically to obtain estimates of the model parameters. The model parameters include the probabilities of particular test results for a woman with given *p53* status, and the prevalence of the various *p53* genotypes in the sample. Optionally, correlations between the test errors for different methods can also be incorporated. Further technical details of the latent class model approach are given elsewhere [6], [11].

Our Model 1 uses the results from the four main laboratory methods used in the study (DHPLC, DBH, RFLP-1, RFLP-2) without assuming any of them to be more or less accurate than the others. The sensitivity and specificity of each test is estimated, relative to the true *p53* status. Model 2 augments the data used in Model 1 by additionally incorporating the cytology results. It examines the relationship between the true *p53* status (which is again the latent variable) and cytology. The effect of the true *p53* status is evaluated through the two-year cumulative risk of SIL for each *p53* value, and by the odds ratios of SIL between pairs of *p53* values. Both Models 1 and 2 assume that the accuracy of the testing methods is independent of a woman’s cytology results. This assumption is later relaxed in a further analysis, as described below in the section, “Analyses of robustness”.

For comparison with the LCM findings, we consider the empirical data, but without invoking a latent variable. For each method, the empirical approach examines the observed *p53* status and its association with the cytology outcome, ignoring the possibility of errors in the laboratory results. The association with cytology is assessed for each test method separately, using the empirical OR between the apparent genotype and cytology.

Finally, we executed a similar series of models to examine test accuracy using the subset of women for whom two additional test results (using the AS-PCR and DS methods) were available.

### Analyses of Robustness

To check the robustness of our model results and examine model assumptions, several secondary analyses were carried out. First, Model 1 was repeated after excluding cases of SIL from the dataset, and changes in the model estimates were noted. Second, Model 1 was repeated using all women in the study, so that they were not excluded based on their cytology status or number of visits.

Third, Models 1 and 2 were repeated to allow test accuracy to vary by cytology status, with likelihood ratio tests being used to assess the statistical significance of this variation. For completeness, we also fitted Models 1 and 2 with test accuracy constrained to be constant across tests; likelihood ratio tests were again used to test the hypothesis of constant accuracy.

Fourth, Models 1 and 2 assume that the tests are conditionally independent, given the true *p53* status of an individual. To test this assumption, we fit extended LCMs that added correlation terms to represent the conditional associations of pairs of test results for women in each of the *p53* groups. Likelihood ratio tests were used to evaluate if the model fit was significantly improved by the inclusion of these correlations.

Finally, two further sets of analyses were carried out with the *p53* polymorphism being re-grouped into only two categories (first, into *Arg/Arg* vs. others, and second, into *Pro/Pro* vs. others, these being consistent with the expectation of risk associations as reported in the literature [2]). This was done because although the total sample size in this study was substantial, when polymorphism status was analysed at three levels for each test, some cross-classified cell frequencies were nevertheless small (particularly when the cytology result was included in the model), leading to some model instability and convergence problems. Aggregation of the data into fewer test levels alleviated many of these difficulties, while still providing useful results.

## Results

Our analyses were conducted with the R statistical package [17] and the software program *LEM* [18]. *R* was used to examine test agreement while *LEM* was used to run all of the models, including both the LCMs and the empirical models that do not allow for test inaccuracy.

### Agreement of Test Results

Table 1 shows cross-tabulations of the *p53* results from each pair of the 4 main laboratory tests, subdivided by cytology status, and with *p53* taken as a variable with 3 levels for the test result. Although the majority of these pairwise test results are concordant, discrepancies were not uncommon.

**Table 1:** Cross tabulation of genotyping test results across four laboratory methods and by cytology status.

		Cytology Status							
		Negative Cytology (SIL = 1)				Any Grade SIL (SIL = 2)			
		Second Test Rating				Second Test Rating			
	First Test Rating	Arg/Arg	Pro/Arg	Pro/Pro	Missing	Arg/Arg	Pro/Arg	Pro/Pro	Missing
First test: DHPLC	Arg/Arg	308	5	8	0	31	0	1	0
Second test: DBH	Pro/Arg	9	365	9	0	1	21	1	0
	Pro/Pro	3	4	139	0	0	0	6	0
First test: DHPLC	Arg/Arg	299	15	7	0	30	1	1	0
Second test: RFLP-1	Pro/Arg	18	360	5	0	1	21	1	0
	Pro/Pro	5	15	126	0	0	1	5	0
First test: DHPLC	Arg/Arg	258	49	13	1	29	1	2	0
Second test: RELP-2	Pro/Arg	9	361	13	0	0	22	1	0
	Pro/Pro	4	2	140	0	0	0	6	0
First test: DBH	Arg/Arg	302	16	2	0	31	1	0	0
Second test: RFLP-1	Pro/Arg	16	355	3	0	0	21	0	0
	Pro/Pro	4	19	133	0	0	1	7	0
First test: DBH	Arg/Arg	262	50	7	1	29	2	1	0
Second test: RFLP-2	Pro/Arg	7	354	13	0	0	21	0	0
	Pro/Pro	2	8	146	0	0	0	8	0
First test: RFLP-1	Arg/Arg	266	46	9	1	28	2	1	0
Second test: RFLP-2	Pro/Arg	3	365	22	0	1	21	1	0
	Pro/Pro	2	1	135	0	0	0	7	0

Table 2 summarises the pairwise test data in terms of crude and chance corrected agreement levels. The crude agreement was 90% or better in all cases, while the weighted kappa statistics ranged between 0.84 and 0.92. While this pattern is encouraging overall, the disagreements between pairs of tests that occurs for approximately 10% of women is clearly problematic. Furthermore, when more than two tests are done, the chance of disagreement between any two tests increases substantially.

**Table 2:** Pairwise comparison of agreement among laboratory methods using crude agreement percentages and kappa coefficients.

		Kappa Coefficient (ASE)		
Laboratory methods compared	Crude Agreement (%)		Unweighted	Weighted
DHPLC and DBH	0.95		0.93 (0.01)	0.92 (0.04)
DHPLC and RFLP-1	0.92		0.88 (0.01)	0.88 (0.04)
DHPLC and RFLP-2	0.90		0.84 (0.02)	0.84 (0.04)
DBH and RFLP-1	0.93		0.89 (0.01)	0.9 (0.04)
DBH and RFLP-2	0.90		0.84 (0.02)	0.86 (0.04)
RFLP-1 and RFLP-2	0.90		0.85 (0.02)	0.85 (0.04)

### Accuracy of Laboratory Test Methods

Table 3 shows the results of the latent Model 1 fitted to the available data from four main tests (DHPLC, DBH, RFLP-1, and RFLP-2). Each cell in the table shows the estimated classification probability for each of the three possible genotype test results (i.e., *Arg/Arg, Arg/Pro, Pro/Pro*), for each laboratory test and for each of the three possible genotype status levels. For instance, women who are truly *Arg/Arg* are estimated to have a 96.4% chance of being classified as *Arg/Arg* by the DHPLC method, a 2.8% chance of being classified as *Arg/Pro*, and a 0.8% chance of being classified as *Pro/Pro*.

**Table 3:** Classification probabilities (standard errors) for p53 genotype by four laboratory tests, according to true p53 status: results from Model 1.

		True p53 status (X)		
Test	Probability	Arg/Arg	Arg/Pro	Pro/Pro
	P(Arg/Arg|X)	0.964(0.010)	0.006(0.005)	0.040(0.016)
DHPLC	P(Arg/Pro|X)	0.028(0.009)	0.994(0.005)	0.037(0.015)
	P(Pro/Pro|X)	0.008(0.005)	0.000*	0.923(0.021)
	P(Arg/Arg|X)	0.997(0.009)	0.011(0.005)	0.000(0.000)
DBH	P(Arg/Pro|X)	0.020(0.006)	0.978(0.008)	0.026(0.013)
	P(Pro/Pro|X)	0.006(0.004)	0.012(0.006)	0.974(0.013)
	P(Arg/Arg|X)	0.957(0.011)	0.023(0.008)	0.013(0.009)
RFLP-1	P(Arg/Pro|X)	0.037(0.010)	0.977(0.008)	0.102(0.024)
	P(Pro/Pro|X)	0.006(0.004)	0.000*	0.886(0.025)
	P(Arg/Arg|X)	0.839(0.020)	0.000*	0.006(0.006)
RFLP-2	P(Arg/Pro|X)	0.138(0.018)	0.978(0.008)	0.014(0.010)
	P(Pro/Pro|X)	0.023(0.008)	0.022(0.008)	0.980(0.011)

* Boundary solution: estimated values of zero for probability and its standard error.

For each of the tests, the model suggests that the most likely classification of any given woman is to her correct *p53* status. To clarify, the LCM estimates the probability that a woman’s true status is each of: *Arg/Arg*, *Arg/Pro* and *Pro/Pro*. The estimate with the highest probability, conditional on her true status, is the most likely test classification for a woman with that true status. We found that across the entire sample, the estimated true status is equal to the most likely test classification. Furthermore, the overall accuracy is reasonably high. The accuracy is high for all three *p53* genotypes for tests DHPLC and DBH. For test RFLP-1, performance is slightly inferior to the other tests for women who are truly *Pro/Pro*; they have approximately a 10% chance of being incorrectly classified as *Arg/Pro*, and an accuracy of only 89% with this method. For test RFLP-2, the classifications are less accurate for *Arg/Arg* women, for whom there is an accuracy of only 84%; women in this group may be classified as *Arg/Pro* with probability approximately 14%. Note that all of the standard errors for these estimated probabilities are small, mostly in the range of 1–2%. However, some caution should be exercised in interpreting these results, because a few model parameters were on a boundary, with an associated zero probability of misclassification for certain data combinations (e.g. the incorrect classification of *Arg/Pro* women into the *Pro/Pro* category by test DHPLC).

It is reassuring that the most likely classification of a given woman by a laboratory test is to her true *p53* genotype status. Moreover, it is interesting to note that the *least* likely classification for homozygous *Arg/Arg* women is to its most dissimilar result (*Pro/Pro*), and *vice versa*, with intermediate probabilities for the heterogeneous *Arg/Pro* group.

### Genotype – Cytology Relationship

Table 4 shows estimates of the cumulative two-year risk of SIL for each level of *p53*, based on Model 2. Also shown are the associated ORs and standard errors from this LCM, for the *Arg/Arg* and *Arg/Pro* groups against the *Pro/Pro* reference category. The results indicate that the homozygous *Arg/Arg* women have the highest risk, with an OR of 1.89, while women in the heterozygous *Arg/Pro* group have an OR that is elevated only slightly above 1. It is important to note again that these parameters are maximum likelihood estimates, and take the possibility of laboratory errors into account. These ORs avoid the ambiguity associated with different empirical OR values from different methods, as discussed below. In addition to the parameters shown in Table 4, this model also gave estimates of test accuracy. Those results were very similar to those from Model 1, so details are not shown here.

**Table 4:** Cumulative 2-year risk (standard errors) of SIL by p53 genotype: results from Model 2.

p53 genotype	SIL risk (SE)	OR (CI*)
Arg/Arg	0.090(0.015)	1.89(0.38, 3.40)
Arg/Pro	0.054(0.011)	1.09(0.19, 1.99)
Pro/Pro	0.050(0.017)	reference

* 95% confidence interval.

Table 5 shows the empirical prevalences of each level of *p53* in the case and non-case groups of the study across the four laboratory tests. Also shown are the empirical ORs for each test, for *Arg/Arg* and *Arg/Pro* against the referent *Pro/Pro* group. The results are quite consistent with those of the LCM (see Table 4), but there is some variation among tests in their estimated prevalences and OR values which would lead to ambiguity if only the empirical results were calculated. For example, the *Arg/Arg* genotype was associated with ORs around 2 for each test, except for DHPLC which had a somewhat higher value (OR of 2.43). The ORs for the *Arg/Pro* group were lower; the DHPLC, RFLP-1 and RFLP-2 methods had values close to 1, but the DHPLC method showed an approximately 50% increase in SIL risk (OR of 1.46) associated with the *Arg/Pro* genotype. When compared with the LCM based estimate from Table 4 (OR of 1.09) one might suspect that the DHPLC method is least accurate and presents the most biased results when considered in isolation.

**Table 5:** Prevalence (standard error) of p53 genotypes in case and non-case groups, and empirical ORs, for four laboratory tests.

		Prevalence (SE)		
p53 genotype	Test	Cases	Non-cases	OR (CI)
Arg/Arg	DHPLC	0.525(0.064)	0.378(0.017)	2.43(0.25–4.61)
	DBH	0.525(0.064)	0.377(0.017)	1.95(0.40–3.50)
	RFLP-1	0.509(0.064)	0.379(0.017)	1.90(0.29–3.51)
	RFLP-2	0.475(0.064)	0.319(0.016)	1.97(0.44, 3.50)
Arg/Pro	DHPLC	0.377(0.062)	0.451(0.017)	1.46(0.13, 2.79)
	DBH	0.344(0.061)	0.440(0.017)	1.10(0.18, 2.02)
	RFLP-1	0.377(0.062)	0.459(0.017)	1.16(0.16, 2.16)
	RFLP-2	0.377(0.062)	0.485(0.017)	1.03(0.21, 1.85)
Pro/Pro	DHPLC	0.098(0.038)	0.172(0.013)	reference
	DBH	0.131(0.043)	0.184(0.013)	reference
	RFLP-1	0.115(0.041)	0.162(0.013)	reference
	RFLP-2	0.148(0.045)	0.196(0.014)	reference

### Extension to 6 Test Methods

Table 6 shows the Model 1 results for the two additional laboratory tests (AS-PCR and DS) that were used less frequently in this study (only for the subset of cases with sufficient DNA material for expanded genotyping assays). The data for the four earlier tests were also included in this analysis, giving a total of six possible tests for a given woman. The accuracy estimates for the four earlier tests were very similar to those shown already, so these numerical details are omitted from Table 6.

**Table 6:** Classification probabilities (standard errors) for p53 genotype by two additional laboratory tests, according to true p53 status: results from Model 1 with extended dataset.

		True p53 status: X (SE)		
Test		Arg/Arg	Arg/Pro	Pro/Pro
AS-PCR	P(Arg/Arg|X)	0.915(0.037)	0.044(0.030)	0.071(0.048)
	P(Arg/Pro|X)	0.052(0.029)	0.852(0.052)	0.138(0.064)
	P(Pro/Pro|X)	0.033(0.023)	0.104(0.044)	0.791(0.076)
DS	P(Arg/Arg|X)	0.895(0.041)	0.054(0.030)	0.000*
	P(Arg/Pro|X)	0.105(0.041)	0.946(0.030)	0.101(0.055)
	P(Pro/Pro|X)	0.000*	0.000*	0.899(0.055)

* Boundary solution: estimated values of zero for probability and its standard error.

The results of Table 6 show that, like the previous four tests, AS-PCR and DS have their highest classification probabilities associated with the actual *p53* value for a given woman, but their accuracy appears to be somewhat lower than for the other four tests. For example, a woman who is *Arg/Arg* has a 92% probability of correct classification by the AS-PCR test, and a 90% probability with the DS test, compared to 96%, 97%, and 96% associated with DHPLC, DBH and RFLP-1 respectively. Similarly, the correctness rates for *Pro/Pro* women (79% for AS-PCR and 90% for DS) are inferior to the earlier tests. As seen with the other tests, AS-PCR and DS also have intermediate misclassification probabilities associated with the heterogeneous group, and the smallest misclassification probabilities for *Arg/Arg* women being classified into *Pro/Pro* and *vice versa*. Overall, the results suggest that AS-PCR and DS are somewhat less accurate than any of the four previous methods, but in light of the restricted dataset in which these comparisons were made it would not be prudent to make generalizations concerning assay accuracy. Note that some of the estimated probabilities in Table 6 are zero, indicating that further boundaries have been encountered. This may imply that the model parameters and their standard errors may be somewhat inaccurate, especially considering the smaller number of women with six test results available, and the correspondingly larger number of parameters required by this extended model.

### Robustness of Results

Given the difficulty of obtaining reliable estimates of accuracy specific to the SIL cases, we ran additional models after excluding them, to assess the robustness of the main results. These additional models yielded test accuracy values that were very similar to those when the SIL cases were included, such as in Table 1, and we therefore conclude that the inclusion of the SIL cases made no material difference to the results. Similarly, when all women were included, regardless of cytology status or number of visits, the results were again very similar, indicating no important impact of these exclusion criteria.

When Model 1 was extended by additionally allowing the test accuracy parameters to depend on cytology status, it failed to converge with the available data, and thus it did not provide estimates for the accuracy parameters. However, we do not expect genotype test accuracy to depend on cytology, especially when considering that one’s genotype may have been measured before abnormal cytology results were documented during follow-up. When Models 1 and 2 were constrained to have constant accuracy parameters across test methods, there was a significant deterioration in the fit to the data (relative to the corresponding unconstrained model), indicating that an assumption of equal test accuracy was untenable.

The extended models that allowed for possible correlations between test errors typically showed no significant improvement in the model fit. The one exception was the suggestion of an interaction between the DHPLC and DBH methods. However, we had no *a priori* reason to expect a correlation between this particular pair of tests. Bearing in mind that there are 6 pairwise correlations between the 4 tests, a multiple comparisons adjustment to the p-value of the DHPLC-DBH association renders it non-significant. Overall we conclude that there is no strong evidence for correlated test errors, and that the results from models assuming conditional independence are valid.

When the analyses were repeated using the *p53* latent variable at only 2 levels (*Arg/Arg* vs. *Arg/Pro* and *Pro/Pro* combined, or *Pro/Pro* vs. *Arg/Arg* and *Arg/Pro* combined) the corresponding estimates of classification probabilities were very similar to those shown in Table 3, but with the advantage that no parameters hit their boundaries of an estimated zero probability. This suggests that although several of the parameters fall on the boundary in the Model 1 results with three levels for *p53*, the other estimates are trustworthy, despite the greater demands on the data by a model with three levels for *p53* as opposed to only two.

## Discussion

Since 1998 [19], there have been numerous studies examining the association between *p53* codon 72 genotype (rs1042522) and risk of cervical cancer or precancer. Although the association is biologically plausible because of the different binding affinities to the E6 gene of HPV by the resulting *Arg* and *Pro* products, there has been considerable heterogeneity in risk estimates across studies [1], [2]. At least some of the heterogeneity may have been due to variations in accuracy and reproducibility of the different assays available to genotype this polymorphism, which prompted us to examine the issue using a data modelling approach in a study in which multiple assays were used to genotype *p53*. Our work in examining the variability in genotyping methods was prompted by the state of uncertainty in the literature at the time. More recent work indicated that although plausible the association may not be real or may be too small to be of clinical relevance [2]. Furthermore, the issue of population stratification, in which race confounds the relationship between p53 status and the outcome, could bias our effect estimates. As previous studies have found that allele frequencies vary across ethnic groups and that the risk of cervical cancer varies by ethnicity it could be the case that population stratification may have biased the effect estimate. Among the three primary racial groups, we found very little difference in the risk of the outcome (risk varied between 8 and 10%) and preliminary analyses of the same data showed that race and age-adjusted effect estimated did not vary importantly from crude estimates (data not shown). However, irrespective of the true nature of the relation between p53 codon 72 polymorphism and cervical neoplasia, we believe that the LCM methodology reported here is a useful tool to examine the relation between disease and a biomarker that is measured with error.

The main advantage of applying the LCM approach to this dataset is that it provides detailed estimates of the accuracy of each laboratory method. In particular, the models yield estimates of the probability of correct or incorrect classification of the *p53* genotype, from each level of the actual genotype variable to each possible observed value, for each test. We are therefore able to assess and compare the performance of each method, but without incorrectly assuming any one of the tests to be a reference or gold standard.

In this study we found that each method had the highest probability of classifying a given woman into her actual *p53* category; the least likely misclassifications were from actual *Arg/Arg* into apparent *Pro/Pro*, and *vice versa.* However, the tests did vary significantly in their accuracy. In our analysis, the DHPLC and DBH laboratory methods were very accurate across levels of p53 status, while the RFLP-1 and RFLP-2 methods were slightly inferior as they misclassified women of a particular p53 status more than 10% of the time.

We also tried to investigate if test accuracy depended on whether a woman had abnormal cytology or not. The number of SIL cases was relatively small, which limited our capability to demonstrate such an effect, if it exists; however, it is reasonable to assume that test accuracy does not depend on cytology, especially considering that abnormalities may not have been present when the genotype was measured.

The LCM analyses also provide estimates of the OR between the *p53* genotype and cervical abnormalities, taking possibility of genotype measurement errors into account. These ORs are based on the ensemble of available test information for each woman, and, being based on maximum likelihood principles, enjoy asymptotic unbiasedness and optimally maximal precision for a correctly specified model. In contrast, the empirical estimates of the OR are based on the subset of data from each method in turn. The empirical test-specific estimates inevitably vary (e.g. the DHPLC test shows substantially higher ORs than the other frequently used tests), and so it is not immediately obvious which test would be preferred in order to convey the risk associated with the *p53* genotypes. The empirical OR estimates were slightly less precise than the corresponding LCM values, because the former are based on a more limited portion of the data than the LCM, which exploits the information from all available tests simultaneously.

Failure of the model to converge when test accuracy was allowed to depend on the cytology result probably reflects the very limited number of SIL cases, because a much larger number of parameters is then required in the extended model. While we are unable to say unequivocally if test accuracy depends on cytology, given the type of specimens used in this study (DNA extracted from exfoliated cervical cells of asymptomatic women with at most low volume lesions) and its cohort design (lesions detected mostly at time points that were different than when the genotyping specimen was obtained), we believe it is reasonable to assume that test accuracy is actually independent of cytology.

The methods we have employed on the Ludwig-McGill study data are quite general, and could be applied in other situations where a gold standard measurement of a risk marker (genetic or otherwise) is not possible. Application of the basic LCM requires that three or more independent tests be available for each subject [6], [20]; in this study we had at least four tests per woman.

The basic model assumes that the test errors are conditionally independent, given the true (but unobserved) status of an individual, which is a plausible expectation. If more than three tests are available, the assumption can be tested by additionally including correlation terms in the LCM, and then assessing the improvement in fit through a likelihood ratio test. Although the assumption of conditionally independent errors has been raised as a concern in the application of the LCM method, a number of investigations [21]–[23] have shown that the assumption may sometimes actually be a reasonable one, or that the model is robust to misspecification in this regard. In the hypothetical situation of a general pattern of positive correlation among tests, the analysis would overestimate test accuracy both in terms of sensitivity and specificity. However, in our earlier work on *p53* [4] we found no evidence of such correlations between the test results for the same woman, using the same method in different laboratories. The likelihood of such correlations between different laboratory methods is presumably smaller, and indeed the analysis in this paper revealed no convincing evidence of correlated errors.

Overall, latent class models are useful for assessing the accuracy of various test procedures without assuming a gold standard. In addition, these methods can provide an adjusted, unbiased estimate of the odds ratio. While each test on its own is imperfect, the adjusted OR estimated by the LCM accounts for this test inaccuracy by taking advantage of the information presented by having an ensemble of testing methods upon which to base its result.

## References

[1] Koushik A, Platt RW, Franco EL (2004) p53 Codon 72 Polymorphism and Cervical Neoplasia A Meta-Analysis Review. Cancer Epidemiology Biomarkers & Prevention 13: 11–22. 10.1158/1055-9965.epi-083-3 14744727

[2] Klug SJ, Ressing M, Koenig J, Abba MC, Agorastos T, et al (2009) *TP53* codon 72 polymorphism and cervical cancer: a pooled analysis of individual data from 49 studies. The lancet oncology 10: 772–784. 19625214 10.1016/S1470-2045(09)70187-1

[3] Glauser W (2010) Standardization of genetic tests needed. Canadian Medical Association Journal 182: E705–E706. 20855485 10.1503/cmaj.109-3669 PMC2952022

[4] Walter SD, Franco EL (2008) Use of latent class models to accommodate inter-laboratory variation in assessing genetic polymorphisms associated with disease risk. BMC genetics 9: 51. 18691419 10.1186/1471-2156-9-51 PMC2536667

[5] Kaldor J, Clayton D (2006) Latent class analysis in chronic disease epidemiology. Statistics in Medicine 4: 327–335. 10.1002/sim.4780040312 3877331

[6] Walter SD, Irwig LM (1988) Estimation of test error rates, disease prevalence and relative risk from misclassified data: a review. Journal of clinical epidemiology 41: 923–937. 3054000 10.1016/0895-4356(88)90110-2

[7] Espeland MA, Handelman SL (1989) Using latent class models to characterize and assess relative error in discrete measurements. Biometrics: 587–599. 2765639

[8] Szatmari P, Volkmar F, Walter S (1995) Evaluation of diagnostic criteria for autism using latent class models. Journal of the American Academy of Child & Adolescent Psychiatry 34: 216–222. 7534756 10.1097/00004583-199502000-00017

[9] Formann AK, Kohlmann T (1996) Latent class analysis in medical research. Statistical methods in medical research 5: 179–211. 8817797 10.1177/096228029600500205

[10] Lau TS (1997) The latent class model for multiple binary screening tests. Statistics in Medicine 16: 2283–2295. 9351165 10.1002/(sici)1097-0258(19971030)16:20<2283::aid-sim658>3.0.co;2-t

[11] Hui SL, Zhou XH (1998) Evaluation of diagnostic tests without gold standards. Statistical methods in medical research 7: 354–370. 9871952 10.1177/096228029800700404

[12] Franco E, Villa L, Rohan T, Ferenczy A, Petzl-Erler M, et al (1999) Design and methods of the Ludwig-McGill longitudinal study of the natural history of human papillomavirus infection and cervical neoplasia in Brazil. Revista Panamericana de Salud Pública 6: 223–233. 10572472 10.1590/s1020-49891999000900001

[13] Schlecht NF, Kulaga S, Robitaille J, Ferreira S, Santos M, et al (2001) Persistent human papillomavirus infection as a predictor of cervical intraepithelial neoplasia. JAMA: the journal of the American Medical Association 286: 3106–3114. 11754676 10.1001/jama.286.24.3106

[14] Rabachini T, Trottier H, Franco E, Villa L (2010) Validation of dot blot hybridization and denaturing high performance liquid chromatography as reliable methods for TP53 codon 72 genotyping in molecular epidemiologic studies. BMC Genetics 11: 44 doi:10.1186/1471-2156-11-44. 20504317 10.1186/1471-2156-11-44 PMC2891603

[15] Cicchetti DV, Allison T (1971) A new procedure for assessing reliability of scoring EEG sleep recordings. American Journal of EEG Technology.

[16] Fleiss JL, Levin B, Paik MC (2003) Statistical Methods for Rates and Proportions: John Wiley & Sons.

[17] Team RDC (2011) R: A language and environment for statistical computing. Vienna, Austria.

[18] Vermunt JK (1997) LEM: A General Program for the Analysis of Categorical Data.

[19] Storey A, Thomas M, Kalita A, Harwood C, Gardiol D, et al (1998) Role of a p53 polymorphism in the development of human papilloma-virus-associated cancer. Nature 393: 229–234. 9607760 10.1038/30400

[20] Walter S (1984) Measuring the reliability of clinical data: the case for using three observers. Revue d’épidémiologie et de santé publique 32: 206. 6522735

[21] Torrance-Rynard VL, Walter SD (1997) Effects of dependent errors in the assessment of diagnostic test performance. Statistics in Medicine 16: 2157–2175. 9330426 10.1002/(sici)1097-0258(19971015)16:19<2157::aid-sim653>3.0.co;2-x

[22] Albert PS (2009) Estimating diagnostic accuracy of multiple binary tests with an imperfect reference standard. Statistics in Medicine 28: 780–797. 19101935 10.1002/sim.3514 PMC2754820

[23] Chu H, Chen S, Louis TA (2009) Random effects models in a meta-analysis of the accuracy of two diagnostic tests without a gold standard. Journal of the American Statistical Association 104: 512–523. 19562044 10.1198/jasa.2009.0017 PMC2701906

